# Dutch Guideline on Knee Arthroscopy Part 2: non-meniscus intra-articular knee injury: a multidisciplinary review by the Dutch Orthopaedic Association

**DOI:** 10.1080/17453674.2020.1850081

**Published:** 2020-11-24

**Authors:** Sander Koëter, Tony G Van Tienen, Paul C Rijk, Patrick W J Vincken, Michiel J M Segers, Bert Van Essen, Nicky Van Melick, Bernadine H Stegeman, Ewoud R A Van Arkel

**Affiliations:** a Department of Orthopedic Surgery Canisius Wilhelmina Ziekenhuis , Nijmegen ;; b Department of Orthopedic Surgery Via Sana , Mill ;; c Department of Orthopedic Surgery Medisch Centrum Leeuwarden , Leeuwarden ;; d Department of Radiology Alrijne Hospital , Leiderdorp ;; e Department of Surgery Sint Antonius Ziekenhuis , Utrecht ;; f Department of Sports Medicine Maxima Medisch Centrum , Veldhoven ;; g Knee Expert Center , Eindhoven ;; h Kennisinstituut van de Federatie Medisch Specialisten , Utrecht ;; i FocusKliniek Orthopedie Haaglanden Medisch Centrum , Den Haag , The Netherlands

## Abstract

Background and purpose — A guideline committee of medical specialists and a physiotherapist was formed on the initiative of the Dutch Orthopedic Association (NOV) to update the Guideline Arthroscopy of the Knee: Indications and Treatment 2010. This next Guideline was developed between June 2017 and December 2019. In part 1 we focused on the meniscus; this part 2 addresses all other aspects of knee arthroscopy.

Methods — The guideline was developed in accordance with the criteria of the AGREE instrument (AGREE II: Appraisal of Guidelines for Research and Evaluation II) with support of a professional methodologist from the Dutch Knowledge Institute of Medical Specialists. The scientific literature was searched and systematically analyzed. Conclusions and recommendations were formulated according to the Grading of Recommendations Assessment, Development, and Evaluation (GRADE) method. Recommendations were developed considering the balance of benefits and harms, the type and quality of evidence, the values and preferences of the people involved, and the costs.

In this part 2 we focus on anterior knee pain, patellar tendinopathy, the role of arthroscopy in the osteoarthritic knee, arthroscopy and patellar dislocation, and osteochondral fractures, and additionally ask what the role is of arthroscopy in bacterial arthritis or ligamentous injury of the knee, and whether arthroscopy is supplemental in the treatment of tibial plateau fractures. We did not address the role of arthroscopy in the discoid meniscus and osteochondritis dissecans in children.

The guideline is published online, in Dutch, and is available from the Dutch Guideline Database (https://richtlijnendatabase.nl/?query=Artroscopie+van+de+knie+&specialism=)

Like most musculoskeletal injuries, knee injuries can be painful and debilitating. Most knee injuries occur during activities of daily living or while participating in sports. In the Netherlands 17% of patients with knee complaints are referred to an orthopedic surgeon (Wagemakers 2010). The most frequent indication for arthroscopy is (suspected) meniscal injury, but other causes of persistent knee complaints may also necessitate arthroscopic surgical treatment. Technical advances in both diagnostic modalities and surgical possibilities as well as shifting insights on indications warrant the necessity for the guideline to address meniscal and non-meniscal injury.

7 clinical questions on non-meniscal related intra-articular pathology of the knee were formulated by a steering group of the Dutch Orthopedic Association (see Guideline recommendations below).

This guideline aims to provide a uniform policy for the care of patients with knee disorders that could possibly be treated with an arthroscopic procedure.

It is written for orthopedic surgeons, sports medicine specialists, physiotherapists, radiologists, and trauma surgeons who are involved in the care of patients with (acute) knee injuries. In addition, this guideline is intended to inform healthcare providers who are also involved in the care of these patients, including pediatricians, rehabilitation doctors, general practitioners, physician assistants, and nurse practitioners.

## Funding and potential conflicts of interest

The guideline development was financially supported by the Dutch Orthopaedic Association (NOV), using governmental funding from the Quality Foundation of the Dutch Association of Medical Specialists in the Netherlands. The authors declare that there is no relevant conflict of interest.

## Method

See Dutch Guideline on Knee Arthroscopy, Part 1 (van Arkel et al. 2020).

**Guideline recommendations according to 7 clinical questions, for literature reviews, see Supplementary data**

### 1. What is the value of arthroscopy in patients with anterior knee pain (AKP), apexitis patella (Jumper’s knee) or patellar tendon tendinopathy?

#### Recommendation

•Do not perform arthroscopy in patients with AKP, because there is no difference in level of pain or function in patients with AKP after arthroscopy compared with nonoperative treatment. In patients with apexitis patella or patellar tendon tendinopathy, most patients do well with nonoperative treatment, but there was a positive effect of arthroscopic shaving compared with nonoperative treatment on level of pain.

### 2. Is there a role for arthroscopy of the knee in patient with osteoarthritis?

#### Recommendation

•Do not perform an arthroscopy in patients with osteoarthritis of the knee with or without debridement or lavage except if the knee is locked due to a sizable loose fragment in the knee.

### 3. Is arthroscopy indicated after patellar dislocation?

#### Recommendation

•Do not perform an arthroscopy in patients in the acute phase after a patellar dislocation; only consider an arthroscopy in case of osteochondral fracture.

### 4. Is arthroscopy indicated for treatment of (osteo)chondral fracture?

#### Recommendation

•Do not perform a diagnostic arthroscopy in patients with a suspected chondral lesion. Consider an arthroscopy in the treatment of an osteochondral fracture.

### 5. Is there a role for arthroscopy in the case of septic arthritis of the knee?

#### Recommendation

•An arthroscopic treatment of septic arthritis combined with systemic antibiotics provides a good treatment option.

### 6. Is arthroscopy indicated for ligamentous injury of the knee?

#### Recommendation

•Do not perform a diagnostic arthroscopy in patients with suspected ligamentous injury.

### 7. Is arthroscopy helpful in the treatment of tibial plateau fracture?

#### Recommendation

•Arthroscopy can have added value in the treatment of unicondylar tibial plateau fracture.

## Discussion

For each question, the scientific level of evidence on which the conclusion was based was graded using the 4 levels of evidence of the GRADE approach (Schünemann et al. 2013). RCTs start with a high level of evidence but must be downgraded if risk of bias (RoB) exists. The RoB tables for RCTs are based on the recommendation made by the Cochrane Collaboration (Higgins et al. 2011). The recommendations given are influenced by many considerations apart from the scientific evidence—such as patient preferences, availability of facilities, or organizational aspects. The recommendations for each question have been based on the scientific evidence, combined with the most important considerations, such as input from the guideline committee experts and feedback from the participating medical societies. The 1st question addresses the role of arthroscopy in patients with anterior knee pain (AKP). The term “anterior knee pain” is a descriptive term that covers all the pain surrounding the patellofemoral joint. It is therefore not a diagnosis in the narrow sense, but a symptom. The working group regarded pain and self-reported knee function as the 2 critical outcome measures. The evidence for this is low grade, because of the small sample size of the included RCT (Kettunen et al. 2007, 2012), which found no difference between the effect of arthroscopy and the effect of nonoperative therapy on level of pain and self-reported function in patients with patellofemoral pain syndrome. In patients with apexitis patella (jumper’s knee) or patellar tendinopathy there was low-grade evidence that there was a positive effect of arthroscopy compared with nonoperative therapy on level of pain in patients with apexitis patella (jumper’s knee) or patellar tendinopathy (Willberg et al. 2011). Due to this low grade of evidence a reticent approach with regard to advising arthroscopy for patients with apexitis is advisable.

The 2nd question regards the relevance of whether arthroscopy of the knee is of value in patients with osteoarthritis, because in older patients a degenerative meniscal lesion can be diagnosed in up to 50% in men in the age range 70–90 years old (Englund et al. 2008), and it can be difficult to differentiate between symptoms caused by the degenerative meniscal injury and symptoms due to early osteoarthritis of the knee. The guideline committee considered self-reported pain scores and self-reported knee function to be critical outcome measures for decision-making. and complications to be an important outcome measure.

It was concluded with moderate-grade evidence that knee arthroscopy did not result in an extra reduction in pain scores or function in the short or long term when compared with nonoperative management in patients with osteoarthritis. The level of evidence was downgraded by 1 level due to serious risk of bias (4 out of 5 trials did not blind the participants, care providers, or outcome assessors) (Brignardello-Petersen et al. 2017). With low-grade evidence it was concluded that arthroscopy may have a small risk of venous thromboembolism and a very small risk of infection compared with nonoperative management in patients with degenerative knee disease. The level of evidence for the outcomes VTE and infections was downgraded for both by 2 levels due to serious risk of bias (used data were not collected for the study) and serious inconsistency (Brignardello-Petersen et al. 2017). To diagnose (early) osteoarthritis of the knee the working group advises making a standing full weight-bearing conventional radiograph in AP, lateral, and fixed flexion in patients over 50 years old. Additional imaging, such as MRI, is necessary only in the absence of osteoarthritis. Arthroscopy in osteoarthritis can be considered when repeated or persistent locking occurs, which is based on engagement of sizable loose fragments in the knee.

The 3rd question addresses the treatment of knee complaints after patellar dislocation. After a patellar dislocation chondral fractures frequently occur; osteochondral fractures are seen less often (Sillanpaa et al. 2008). Chondral and osteochondral fragments can form loose bodies in the knee and arthroscopic removal of the loose bodies is propagated by some surgeons, sometimes combined with other arthroscopic or open operative procedures. This early surgical repair is now more common, without clear evidence to support this approach. We found no recent literature in the databases Medline (via OVID) and Embase (via Embase.com) between 2009 and January 17, 2018 that met the selection criteria. The guideline committee concluded (expert opinion) that only in patients with accompanying osteochondral fractures that can possibly be reattached is arthroscopic or open surgery indicated; in all other cases conservative treatment is the best first treatment option.

The 4th question addresses the indication for arthroscopy in the case of knee complaints caused by chondral or osteochondral fractures in the acute phase. The working group regarded the development of osteoarthritis and response to treatment as critical outcome measures. We found no recent literature that met the selection criteria, the old guideline concerned 3 case series and a dissertation on fixation techniques that are not eligible according to the current selection criteria. The guideline committee advises, based on expert opinion, that arthroscopy is not indicated in case of chondral fracture, but can be considered in the case of refixation of an osteochondral fracture or removal of sizable fragments engaging persistent or recurrent locking.

Concerning the clinical questions 5 to 7 we could not find new literature in our search that we could analyze according to the GRADE criteria; therefore the old text of the guideline Knee Arthroscopy 2010 was adopted.

The 5th question addresses the treatment of ligamentous injury of the knee. Because the treatment of anterior cruciate ligament (ACL) injury is described in a separate guideline (Meuffels et al. 2012), ACL injury is excluded from this guideline. In former years the use of arthroscopy to address concomitant injury in case of hemarthrosis was widespread because the incidence of additional injury is high: in more than 50% of patients 1 or more ligamentous injuries are present. Based on expert opinion, the working group concludes that there is no role for diagnostic arthroscopy. Clinical examination, conventional radiographs (to exclude fractures), and MRI are the diagnostic tools of choice.

The treatment of septic arthritis, addressed in the 6th question, is controversial, and differences persist between clinical specialties (orthopedic surgeons, rheumatologist, family physicians). We found no recent literature that met the selection criteria; all evidence on the treatment of septic arthritis is based on older, retrospective studies. Based on these studies and expert opinion, the working group concludes that arthroscopic debridement of septic arthritis seems to provide favorable results when combined with systemic antibiotics (Stutz et al. 2000, Wirtz et al. 2001). In addition, arthroscopic debridement in the acute phase seems to provide better results than recurrent needle aspiration (Ayral 2005). In the case of persistent septic arthritis arthroscopic debridement can be repeated (Stutz et al. 2000).

In the case of tibial plateau fractures (question 7), arthroscopy was advocated in the literature in the 1990s (Jackson 1995). More recent literature focused on selected fractures (unicondylar fractures type II [split depression], and type III [isolated depression]). In these fracture types arthroscopic-assisted techniques resulted in fewer complications and faster rehabilitation (Ohdera et al. 2003, Musahl et al. 2009). Based on the limited literature and expert opinion the working group concludes that arthroscopic treatment can be indicated in the treatment of unicondylar tibial plateau fracture. Diagnostic arthroscopy is not indicated in the treatment of tibial plateau fracture.

In recent years evidence has accumulated that questions the effectiveness and rationalization of arthroscopy for the treatment of AKP and osteoarthritis. This observation might have induced a more critical appraisal of other indications for arthroscopy, such as after a patellar dislocation, osteochondral fracture, bacterial arthritis, ligamentous injury of the knee, or tibial plateau fracture. This guideline provides evidence-based consideration of the current indications for arthroscopy.

## Supplementary data

Literature reviews are available as supplementary data in the online version of this article, http://dx.doi.org/10.1080/17453674.2020.1850081
